# Immune Repertoires in Various Dermatologic and Autoimmune Diseases

**DOI:** 10.3390/genes15121591

**Published:** 2024-12-11

**Authors:** Hanna Terhaar, Victoria Jiminez, Emily Grant, Camden Collins, Mohamed Khass, Nabiha Yusuf

**Affiliations:** 1 Heersink School of Medicine, University of Alabama at Birmingham, Birmingham, AL 35294, USA; 2 Department of Endodontics, University of Alabama at Birmingham, Birmingham, AL 35294, USA; 3 Department of Dermatology, University of Alabama at Birmingham, Birmingham, AL 35294, USA

**Keywords:** immune repertoires, autoimmune diseases, dermatologic diseases, adaptive immune system, molecular biology

## Abstract

The immune repertoire (IR) is a term that defines the combined unique genetic rearrangements of antigen receptors expressed by B and T lymphocytes. The IR determines the ability of the immune system to identify and respond to foreign antigens while preserving tolerance to host antigens. When immune tolerance is disrupted, development of autoimmune diseases can occur due to the attack of self-antigens. Recent technical advances in immune profiling allowed identification of common patterns and shared antigen-binding sequences unique to diverse array of diseases. However, there is no current literature to date evaluates IR findings in autoimmune and skin inflammatory conditions. In this review, we provide an overview of the past and current research findings of IR in various autoimmune and dermatologic conditions. Enriching our understanding of IRs in these conditions is critical for understanding the pathophysiology behind autoimmune skin disease onset and progression. Furthermore, understanding B-cell and T-cell IR will help devise therapeutic treatments in the hopes of restoring immune tolerance and preventing disease onset and progression.

## 1. Introduction

The immune repertoire (IR) refers to all expressed T cell receptor (TCR) and B cell receptor (BCR) genetic recombination in the adaptive immune system. These genetic rearrangements determine the immune system’s ability to respond to a variety of pathogens [1]. The adaptive immune system differs from the innate immune system in its ability to recognize small differences in antigen epitopes, to the degree of even one amino acid, and to initiate a subsequent immune response. Additionally, the adaptive immune system develops immune memory that boosts the secondary immune response upon encountering the same antigen. The main cellular components of the adaptive immune system are B-cells and T-cells. The formation of TCR and BCR follows the same pattern of genetic recombination of Variable (V), Diversity (D), and Joining (J) gene rearrangement. The expressed repertoire of TCRs and BCRs allows both T-cells and B-cells to recognize diverse array of antigens [2]. T-cells use their TCRs to recognize epitopes bound to major histocompatibility complexes [3]. In contrast, B-cells use their BCRs to capture and present antigens. Binding of BCR to its specific (cognate) epitope initiates a cascade of reactions, such as class switch recombination and somatic hypermutation, leading to the production of high-affinity antibodies and memory cells [3].

The diversity of IR is important for the wide recognition of huge amounts of pathogens. Regulation of repertoire development is critical to avoid initiation of response toward self-antigens and subsequent autoimmunity [4].

### Importance of Studying Immune Repertoire

Understanding the composition of the immune repertoire is essential for prevention and directed therapy for chronic diseases. Over time, dysregulation of the immune response has been associated with decreased ability to mount responses against infections, vaccines, cancer, and autoimmunity [5]. Aging also induces low-grade inflammation (inflammaging) that results in the dysfunction of both innate and adaptive immune cell types. This creates cell-intrinsic defects in lymphocytes, and their signaling which impact other cell types, including stromal cells in both primary and secondary lymphoid organs [5]. Additionally, obesity substantially alters the T cell and B cell repertoires by promoting a state of inflammation while reducing anti-inflammatory immune surveillance. Adipocytes have been observed to increase the secretion of pro-inflammatory molecules, including tumor necrosis factor α (TNF-α), interleukins (IL)–IL-6, and IL-8, resulting in a significant increase of macrophages in adipose tissue [6]. In T-cells, a surplus of glucose and fatty acids, with an overwhelming amount of leptin production, enriches pro-inflammatory subsets, such as Th1 and Th17 cells, while decreases regulatory T cell(Treg) activity and reduces their ability to suppress inflammation [7]. For B-cells, obesity alters antibody production, as high-fat diet promotes the accumulation of selected murine B-cells in visceral adipose tissue [8]. Additionally, insulin resistance promotes B-cell secretion of IgG antibodies, and enhances local innate response from macrophages to produce TNF-α production in an Fc-dependent process This possibly leads to affect insulin signaling molecules as well as other self- or foreign antigens [9]. As a consequence, B-cells exacerbate inflammation through the production of autoantibodies. B cells also cross-talk with select adipose resident macrophages, CD4+ and CD8+ T-cells, and ultimately alter adaptive immune responses while promoting susceptibility to the risk of immune-related diseases [9].

Inborn errors of metabolism (IEI) are also recognized as a cause of B cell and T cell repertoire alteration, ultimately leading to the onset of various autoimmune and dermatologic diseases. In regards to T cell repertoire alteration, impaired central and peripheral tolerance mechanisms observed in IEIs such as autoimmune lymphoproliferative syndrome (ALS) and autoimmune polyendocrinopathy-candidiasis-ectodermal dystrophy (APECED) lead to the persistence of autoreactive T-cells and a harmful T cell repertoire [10]. Additionally, chronic metabolic imbalances and essential substrate deficiencies caused by genetic mutations can alter the thymic and bone marrow microenvironments, affecting T cell selection, B cell differentiation and subsequently germinal center reactions [11]. An example of IEIs, adenosine deaminase deficiency, which predisposes individuals to recurrent infections and autoimmune complications [12].

In terms of cutaneous autoinflammatory and autoimmune conditions, the alteration of immune cells and their molecular signaling patterns have been affected [13]. For example, it has been observed that the repertoire for atopic dermatitis has been associated with increased expression of type 1 and type 2 cytokines and IL-17/IL-22 [13].

Information from immune repertoire analysis has guided the development of many biological treatments, including interleukin antagonists and antibody inhibitors in many diseases such as psoriasis, atopic dermatitis, chronic spontaneous urticaria, and hidradenitis suppurativa [14].

While identifying specific patterns of immune regulation is essential in these types of diseases, elucidating the primary inciting factors before the symptomatic onset of the diseases could significantly prevent clinical manifestations and determine symptom severity. Breaching B-cell tolerance is considered as an initiating factor in autoimmunity, but mechanisms have not yet been thoroughly defined [15]. Interestingly, dysregulated B cell signaling alters BCR repertoire composition and generates high-affinity autoantibodies, as seen in autoimmune disorders such as type I diabetes and systemic lupus erythematosus [15]. With the vastness and diversity of technologies that serve IR analysis, the question is how to use this knowledge in identifying pathogenic variants to prevent the development of autoimmune diseases. Advances in next-generation sequencing have allowed the discovery of molecular patterns that reveal autoreactive BCRs or TCRs [16]. It has been proposed that there is a delicate balance between the need for TCR and BCR diversity that serves recognition of a wide range of foreign antigens and preservation of reactive T- and B cells against autoantigens [17]. Studying IR of autoinflammatory and autoimmune disease can distinguish these implicated antigens which in turn will help monitor disease severity and even evaluate response to therapies [17]. Therefore, the purpose of this review is to evaluate recent studies of shared sequences within the immune repertoire that are found among various dermatologic and autoimmune diseases.

## 2. Methodology

A comprehensive search was conducted in PubMed and Embase to identify all relevant articles published between 1990 and 2024. The search strategy included keywords and MeSH terms related to the B cell and T cell repertoire, autoimmune diseases, and dermatologic conditions. Boolean operators such as “B cell repertoire, T cell repertoire”, “autoimmune disease”, and specific dermatologic conditions such as “vitiligo” and “melanoma” were used to refine the search. The search results were uploaded into Covidence version 2.0, a software tool used to streamline the process of conducting a systematic review. Covidence removed duplicates from the uploaded databases, and a title/abstract screening and full-text screening were conducted in the database. Inclusion criteria included studies published in English or languages other than English that had a reliable translation and focused on B cell or T cell repertoire studies in the context of autoimmune diseases or dermatologic conditions. A final list of 70 studies was included in this review article addresses the relationship between T cell and B cell repertoires and autoimmune and dermatologic conditions.

## 3. Immune Repertoires in Various Autoimmune Diseases

Table 1 and Table 2 summarize the key findings in the Immune Repertoires of various autoimmune and dermatologic diseases.

### 3.1. Ankylosing Spondylitis

Ankylosing Spondylitis (AS), or “Bechterew’s disease”, is a chronic autoimmune disease affecting the joints of the axial skeleton. T cell infiltration is believed to be the mechanism of the attack on the joints. An association with the HLA-B*27 allele has been linked with T cell involvement [18]. Few studies in the literature have explored the T cell repertoire variation in AS. Fourteen motifs have been found to be enhanced in B*27-positive AS patients versus healthy individuals with the B*27-positive allele. In this study, the control population size was relatively small [19]. Eight TCR-CD23 motifs have been expanded in 25 AS patients positive for HLA-B27 in comparison to healthy controls [20]. In 7/8 of the expanded clonotypes, CD8+ T cells were identified [20]. Further studies emphasize the identification of both CD8+ and CD4+ TCR clonotypes with an expansion of a TCR-CD3 motif among AS individuals, particularly cases associated with a positive HLA-B27 allele [21,22]. Clonal expansion and depletion cause dysbiosis within the immune system and allow for the remission and relapse pattern of this disease. Hematopoietic Stem Cell Transplant has been found to be an effective treatment by resetting the levels of T-cells systemically [87].

### 3.2. Autoimmune Encephalomyelitis

Autoimmune encephalitis (AIE) is characterized by inflammation of the brain parenchyma, affecting more than 250,000 patients in the last decade alone in the US [88]. Patients with AIE commonly present with altered mental status, headache, or seizures. AIE with concurrent inflammation of the spinal cord (myelitis) leads to a diagnosis of encephalomyelitis. There are several subtypes of AIEs, which are categorized by the specific antibodies that target unique proteins or receptors in the brain.

The experimental autoimmune encephalomyelitis (EAE) animal model has been well studied to further understand the immune mechanisms underlying the disease [23,89]. One study focused on the potential role of the Nogo-66 receptor 1 (NGR1) in mediating the immunological response to EAE but found minimal impacts on immune cell types [24]. Natural regulatory CD4+CD25+ cells expressing PLP139-151 have been shown to contribute to resistance to EAE [25]. Additionally, the lack of bi-allelic TCR recombination promoted γδ T-cell development, contributing to more severe EAE [26]. More studies are needed to determine the extent of the role of γδ T-cells in EAE progression. Similar BCR with a third complementary determining region (CDR3) length, important for antigen binding, have been shared in different types of antibody-mediated autoimmune encephalitis (mainly anti-NMDARE and anti-LGI1E) [27].

### 3.3. Inflammatory Bowel Disease

Inflammatory bowel diseases (IBDs) are multifactorial disorders affecting the gastrointestinal system, such as Crohn’s disease (CD) or ulcerative colitis (UC). Patients with IBD typically present with abdominal pain, rectal bleeding, and weight loss. IBD is highly prevalent, affecting between 2 and 3 million people in the United States alone.

Patients with IBD experience persistent inflammatory responses to gut bacteria. Patients with mutations in IL10/IL10R have a significant increase in circulating T-cells with shorter CDR3-β length and altered hydrophobicity [28]. Analysis of TCR repertoire in T-cells of IBD patients showed lower clonotype diversity and stronger clonal expansion, which has been attributed to selective bias in V and J gene usage [29]. Inhibitory and activating KIRs have been associated with IBD, specifically KIR2DS1 and KIR2DS5. Imbalance in KIR genes and HLA ligands might also contribute to the diverse pathogenesis observed in IBD [90].

### 3.4. Juvenile Idiopathic Arthritis

Juvenile idiopathic arthritis (JIA) is a chronic inflammatory disease characterized by joint inflammation due to immune dysregulation in children. Patients with JIA present with joint stiffness, pain, and swelling. In the United States, there is an estimated total of 294,000 children affected by JIA [30]. Studies emphasize the abundance of Foxp3+ regulatory cells (Treg) at inflammation sites in JIA patients [31]. B-cells in the synovial fluid of the joints of JIA patients showed CD27 memory B-cells and class-switched B-cells expressing immunoglobulins [32]. JIA patients on immunosuppressive drugs such as methotrexate, prednisone, and other biological therapies modified clonotype distribution by displaying less clonotype stability, increasing fluctuation in clonotype distribution, and more variable IFNy response of the M1_58–66_ specific RS/RA clonotypes in JIA [33]. Studies investigating mediators between the bloodstream and synovium identified a subset of CD4+ T-cells, CPLs that mimic the phenotype of synovial lymphocytes and produce cytokines contributing to the immunological response [34]. In the cases of systemic JIA (sJIA), the most severe form of JIA, CD8+ TCRVβ repertoire, was found to be oligoclonal [91]. Following autologous stem cell transplant for sJIA patients, new TCR sequences were found, suggesting the presence of a chimeric TCR repertoire in sJIA [91].

### 3.5. Multiple Sclerosis

Multiple sclerosis (MS) affects close to 1 million individuals in the United States. MS is a demyelinating, autoimmune disease of the central nervous system characterized by inflammation driven by altered B and T-cell function [35]. Patients with MS commonly suffer from a lack of coordination, blurred or double vision, and numbness. Peripheral CD27+IgD− memory B-cells, CD27hiCD38hi plasma cells/plasmablasts, or CD27−IgD− B-cells have been identified as a potential biomarker of MS [36]. Clonal expansion of B-cells, mostly Ig class-switched and CD27+, has been observed in the CSF, meninges, CNS parenchyma, and draining cervical lymph nodes in MS patients [37,38,92]. The BCR repertoire in MS also showed somatic mutations in the BCR VH region [37]. Impaired maturation of regulatory B cells has been associated with MS as well [93].

The first pathogenic event in MS was hypothesized to be related to disruption in tolerance and subsequent activation of myelin-specific T-cells [94]. Patients with MS have impaired TGFbeta signaling and, hence, disrupted regulatory T cell development [39]. TCRs in brain-responding T-cells are clonal and display little overlap with antigens [40].

### 3.6. Myasthenia Gravis

Myasthenia Gravis (MG) is an autoimmune condition affecting the neuromuscular junction where autoantibodies against acetylcholine receptors (AchR) are generated and result in the inhibition of muscular contraction and muscle weakness [95]. The nicotinic AchR antibody produced by the B-cells is A specific auto antibody B12L was found to be raised against the main immunogenic region (MIR) of the N-terminal extracellular domain of the AchR [41,96]. B-cell populations show a bias to certain IGHV4 family gene usage, specifically in naïve and memory B-cells [42]. Studies have shown that MG patients with AChR and Muscle-Specific Tyrosine Kinase (MuSK) protein antibodies displayed biased gene segment usage in both V_H_ and V_L_ sequences in the naïve and memory compartments [42]. Additionally, the memory compartment of patients with AChR antibodies had a decrease in positive selection of somatic mutations in the V_H_ CDR and altered V_H_ CDR3 physicochemical properties [42]. Autoantibody-producing B cells are developed due to T-cell activation. The Vav1 protein is a signaling molecule responsible for T-cell activation and immune B-cell development. The Vav1R63W variant of this protein has been found to be associated with MG autoimmune neuroinflammation. In mouse models, it has been found that individuals with this variant have a lower level of AchRs and increased pro-inflammatory cytokines such as IL-17A and IFN-γ produced by CD4+ cell production as a result of altered diversity of the TCR repertoire [95]. According to TCR repertoire analysis from the thymus and peripheral blood of MG patients, there are specific T-cell clones associated with the disease. The TRBV12-4, TRBV4-3, and TRBV6-6 gene variants and several VJ gene variants were upregulated in MG patients both in the thymus and blood. Also, T-cells with longer CDR3 sequences were found to be more abundant in the thymus of MG patients. These variants were found to be of higher abundance within T-cell clonality in MG. The role of these variants in disease is to promote the formation of autoantibody-producing B-cells that ultimately cause disease [43]. The thymus is the central organ in immune cell development and can be an integral part of MG. Thymectomy is often recommended in the course of treatment for patients with MG in an attempt to eradicate the autoantibody-producing immune cells. However, even after removal of the thymus, patients may still have circulating disease-causing cells. These patients have more severe presentation and persistence of MG than those without circulating cells present after thymectomy [97].

A smaller subset of MG patients showed different mechanism of disease that includes antibodies specifically targeting the Muscle-Specific Tyrosine Kinase (MuSK) protein, which mediates muscle contraction. Those patients experienced immune mediation through MuSK-specific B cell clonal expansion and this is often treated through B-cell depletion therapy such as Rituximab. However, some patients experience relapse of disease after cessation of Rituximab therapy [98]. Relapse of disease post-Rituximab treatment is due to the re-emergence of persistent B-cell populations such as plasmablasts and long-lived plasma cells. The autoantibodies of the MuSK MG subset are of the IgG4 subclass. These do not activate the complement system like the IgG1 and IgG3 antibodies of the AchR pathogenesis [99].

Myasthenic Crisis (MC) may occur in patients with MG when a rapid deterioration of muscles occurs, specifically affecting the respiratory muscles requiring emergency innervation such as intubation. Hypercytokinemia occurs during MC and causes immune dysregulation. FCGR3B+ monocytes are found to induce significant pro-inflammatory activity during MC. These monocytes are associated with the release of IL-1B and CXCL8 (IL-8), which contributes to the high neutrophil-to-lymphocyte ratio during MC. T and B cell dysregulation during MC exhibit oligoclonal expansion showing signs of exhaustion. Plasma exchange may be a useful therapy during MC to eradicate circulating cytokines and regulate T and B-cell expansion [44].

### 3.7. Neuromyelitis Optica

Neuromyelitis Optica (NMO) is a rare disease of the CNS characterized by severe inflammation and demyelination of the optic nerves and spinal cord. Aquaporin-4 (AQP4) is a water channel protein highly expressed in astrocytes and is the target autoantigen in NMO. AQP4 antibodies are detected in more than 80% of people with NMO. B-cells play a crucial role in NMO by producing anti-AQP4 antibodies and purging AQP4-specific T-cell clones. B-cells express AQP4 endogenously in response to CD40 and IL-21 activation, which allows them to present AQP4 to T-cells with specific TCRs, efficiently removing AQP4-reactive thymic TCR repertoire [45]. Immunogenic T-cell epitopes raised against the N-terminal region of AQP4 have been identified. T-cells shape the specific IgG response pattern associated with a mixed Th1/Th2 T-cell response in NMO [46]. Studies showed that NMO patients have reduced diversity and shorter CDR3 length in their TCRβ repertoire with abnormal VDJ recombination and a greater proportion of nonfunctional TCR clones [47]. Additionally, AQP4-specific Th17 cells have been found to induce paralysis in murine studies, regardless of wild-type or B-cell-deficient status or an innate Thymic AQP4 deficiency, suggesting an essential role in the pathogenesis of NMO [48].

### 3.8. Primary Biliary Cholangitis

Primary Biliary Cholangitis (PBC) is an autoimmune liver disease characterized by injury to the small bile ducts. PBC is more common in women than men, affecting about 65 out of every 100,000 women in the United States. Common symptoms of PBC include fatigue and itching. CD8+ T-cells have been demonstrated to play a key pathogenic role in PBC. Both CD8αα and CD8αβ T-cells are associated with lymphocyte differentiation and cytokine production. Specifically, hepatic CD8αα T-cells are terminally differentiated and display higher cytokine production and cytotoxicity. Furthermore, CD8αα T-cells exhibit greater clonal expansion and less diverse repertoire than CD8αβ T-cells [100].

### 3.9. Rheumatoid Arthritis

Rheumatoid Arthritis (RA) is characterized by synovial inflammation and damage to the bone and surrounding tissues. The disease often involves the activation of B-cells and subsequent production of anti-citrullinated protein bodies (ACPAs) [49,50,101]. These B-cells exhibit more somatic hypermutations and utilize more class-switched isotypes [101]. Studies have found increased miR-155-5p expression in blood-derived CD19+ B-cells in newly diagnosed RA patients and decreased production of CD27+IgD+ B-cells and the IgM antibodies they produce in peripheral blood of RA patients, suggesting a unique BCR repertoire [51,52]. Abnormalities in B-cell tolerance and continuous activation of selected B-cell clones lead to imbalances in antibody levels and further contribute to the pathogenesis of RA [53,54]. Moreover, abnormalities in CD4+ T-cells are also common in RA [55,56,57]. The TCR repertoire in RA showed reduced diversity and increased usage of specific V/J segments [58]. Moreover, the increased expression of DRB1 genes showed an increased contribution to RA predisposition [59]. Finally, Antigen 1 has been characterized with the highest number of T-cell epitopes related to RA [60].

### 3.10. Sjögren’s Syndrome

Sjögren’s Syndrome (SS) is an autoimmune disorder associated with inflammation of the secretory glands, specifically the lacrimal and salivary glands, and significantly affects more women than men. Patients with SS commonly present with dry mouth and dry eyes. Using a primary Sjögren’s Syndrome mouse model (Id3−/−), studies have determined that salivary gland B-cells in SS display increased JH1 usage and have similar CDR-H3 hydrophobicity [61]. Prior studies have also suggested that B cell checkpoints may play a role in the immunological response. While a defect in the early B cell checkpoint does alter development, evidence suggests that it is not sufficient to drive SS in the presence of preformed autoantibodies, and instead, a more diverse B cell repertoire with T-B-cell collaboration is responsible for disease progression [102]. B-cells obtained from cervical lymph nodes have also been shown to produce reactive antibodies against salivary tissue in SS [62]. No differences were found in the IGHV B-cell repertoire of intraductal and periductal B-cells. Closely related sequences between the regions support substantial B cell exchange between the two regions in SS [63]. TCR repertoire analysis of infiltrating T helper-1 (Th-1) cells revealed restricted clonal diversity. Th17 cells, in particular, are prevalent in SS glands, and Th1 cells express a unique CDR3 motif producing IFN-γ, an antiviral cytokine [64]. Finally, with SS exhibiting gender bias, differences in effector T-cell populations in male and female models have been studied. Female models displayed unique motifs in TCR hypervariable regions and alternative selection mechanisms compared to male models of SS [65].

### 3.11. Systemic Lupus Erythematous

Systemic lupus erythematosus (SLE) is an autoimmune disease that causes inflammation and tissue damage to multiple organ systems, including the skin and joints. Over 200,000 people are estimated to have SLE, with women being more likely to develop the disease than men.

Current literature on the immune repertoire of SLE stresses deviations in both T-cell and B-cell dynamics. SLE is characterized by abnormal B-cell activation, with reduced diversity and altered VDJ gene usage and CDR3 sequences [66,103]. A linkage to defects in B-cell tolerance checkpoints, particularly the second tolerance checkpoint, has been speculated as leading to the persistence of autoreactive B-cells [67]. Despite changes in B-cells, NK cells in SLE patients display minimal phenotypic alterations, while T cells show increased clonality [104]. Dysregulated B-cell-intrinsic TLR signaling also contributes to disease propagation [105,106]. Therapeutic approaches targeting B-cell differentiation Btk inhibitors and CAR T-cell therapies have been proposed [107,108]. Btk inhibitors modulated the immune cell repertoire by reducing gene expression of known mediators of kidney damage and fibrosis IL1-F6, Grem1, and LCn2, as well as uniquely downregulating genes associated with B-cell proliferation (Fcr15) [107]. CAR T-cell therapies effectively remodulated the B-cell population by eliminating IgG and IgA memory B-cells and enriching for IgM or IgD cells post-therapy [108].

### 3.12. Systemic Sclerosis

Systemic Sclerosis (SSc) is a fibrotic autoimmune disease marked by excessive deposition of connective tissue and vascular damage throughout the body. This damage leads to scarring and impaired function of various organs. Deposition of fibrotic tissues can be triggered by infection, inflammation, and tissue injury. Patients experience liver cirrhosis, pulmonary fibrosis, and renal interstitial fibrosis as secondary results of the SSc [68]. The immune landscape of these patients is marked by B-cell clonal expansion with altered VDJ recombination and increased low mutation-immunoglobulin D (IgD) positive cells with loads. The average CDR3 in SSc patients was shorter than in non-diseased subjects [109]. B cell depletion drugs like Rituximab show promise in treating this disease [69].

Another key feature of this disease is fibrosis. This is facilitated by CD4+ and CD8+ T-cells interacting with fibroblasts within the skin and creating fibrotic deposits. TCR sequencing has shown specific TCRβ repertoire in CD4+ and CD8+ T-cells, indicating specific autoantigen activation and chronic inflammation [110].

### 3.13. Thyroid Diseases

Thyroid diseases such as papillary thyroid cancer (PTC), Graves Disease, or autoimmune thyroiditis, also known as Hashimoto’s, are greatly influenced by the immune repertoire of the body. Grave’s disease is the most common cause of hyperthyroidism and is caused by thyroid-stimulating antibodies against the thyroid receptors of the thyroid gland [111]. Hashimoto’s is associated with the autoimmune destruction of thyroid follicles and the production of autoantibodies. Tregs are found to be the self-regulating preventative against Hashimoto’s [70]. In PTC patients, CD8+ and CD4+ T-cells were found in approximately equal amounts. Conversely, in patients presenting with PTC and Hashimoto’s, there were significant increases in CD3+, CD4+, and CD8+ T-cells, with the highest levels being in CD4+ as compared to PTC alone [71]. In a study that compared autoimmune thyroiditis with healthy patients, a statistically significant expansion of the TCR immune repertoire of autoimmune thyroiditis patients was found compared to normal subjects [72]. Additionally, specific TCR clones, such as TRBV15, TRBV-2, TRBV9, TRBV3-2, TRBV7-8, TRBV25-1, TRBV12-4, and TRBV27, were found to be preferentially expressed in Graves’ disease patients while TRBV29-1, TRBV12-4, TRBV7-2, TRBV6-5, TRBV9, TRBV27 and TRBV4-2 were found to be more expressed in Hashimoto’s thyroiditis patients [72]. In this same study, patients with refractory Graves’ disease were found to develop a specific immune repertoire that differentiated Grave disease patients from the normal subjects, whereas the differentiation in newly diagnosed Graves’ disease was not as prominent [72]. In another study, a specific TCR motif that detects the autoantigen TPO was not found; however, a negative correlation was found between the average hydrophobicity of amino acids in the CDR3 (N) region of the TCR α chain and the strength of simulation of cross-reactivity [112]. Therefore, the higher-affinity clones responsible for Hashimoto’s thyroiditis can be hypothesized to be associated with a greater degree of hydrophobicity in CDR3 α region amino acids [112].

### 3.14. Type 1 Diabetes Mellitus

Type 1 Diabetes (T1D) is a chronic autoimmune disease that results from the destruction of β cells in the pancreas, leading to an inability to secrete insulin and regulate blood glucose levels.

The frequencies of CD3+, CD4+, and CD8+ T-cells are variable in T1D patients with imbalances between autoreactive and regulatory T-cells in the peripheral blood [73]. Key TCR gene segments such as TRBV13-2, TRBV13-1, and TRBV5 are overrepresented in individuals with T1D [74]. Genetic associations such as the HLA-DR3/DR4-DQ8 haplotype influence the development of the TCR repertoire, and HLA-DQ2 and HLA-DQ8 display links with T1D risk and CD4+ T-cell selection [75,76]. The usage of specific J genes has been identified in insulin-binding BCRs [113], but the BCR repertoire data are still limited.

Because thymic dysfunction in establishing central self-tolerance to insulin-secreting islet β cells in the pancreas is central to T1D, novel therapies have explored negative/tolerogenic self-vaccinations to reprogram tolerance [114]. Additionally, autoantigen-specific immunomodulatory therapy has been supposed to overcome dysfunctional checkpoints in the central tolerance pathway, while islet transplantation has also been explored [115,116]. Furthermore, combination therapies involving anti-CD3 antibodies and GAD65-expressing plasmids have shown promise in reversing new-onset T1D specifically, with effectiveness varying based on MHC types [77].

## 4. Immune Repertoires in Various Dermatologic Diseases

### 4.1. Alopecia Areata

Alopecia Areata (AA) is one of the most common autoimmune diseases which targets growing hair follicles. In AA, immune cells attack hair follicles resulting in hair loss in the scalp but also through the entire body. The immune mechanism of this disease involves cytotoxic T cell expansion. Biopsies of lesional skin showed clonal expansion of CD8+ T-cells, suggesting an antigen-driven process. The dominant effector population responsible for hair loss associated with AA is suggested to be CD8+NKG2D+ cells [78]. Inhibitors of the JAK-Stat pathway have been found to be effective in reducing clonal expansion of CD8+ T-cells. Further research should be conducted to identify more targets and distinct BCR and TCR repertoire [117].

### 4.2. Melanoma

Melanoma is an aggressive skin cancer involving pigment-producing melanocytes. It is caused by exposure to UV radiation from the sun. It is highly metastatic and more aggressive than other skin cancers. Both T and B-cell proliferation have been observed within the tumor microenvironment. B-cells specifically exhibit clonal expansion and class switch recombination. These cells also produce autoantigens showing activity within the tumor microenvironment [79]. Moreover, these tumor-infiltrating B cells produce antibodies against disialylated glycosphingolipids (GD3) expressed by melanoma [80]. The levels of B-cell proliferation will determine tumor progression. The B-cell involvement is highly complex and shows contribution to both tumor immunity and autoimmune reaction [79]. Melanoma patients were found to have an increased expression of the Valpha12.1 chain in the MELOE-1/A2-specific T cells specific for the MELOE-1/2a epitope expressed by tumor cells [81]. Furthermore, studies have found expanded CDR3α and β clonotypes of MELOE-specific T-Cells [118]. Melanoma patients with this specific repertoire have been found to have an increased relapse free survival and, therefore, future research should consider the MELOE-1/2a antigen as a potential immunotherapy target.

### 4.3. Oral Squamous Cell Carcinoma

Oral squamous cell carcinoma (OSCC) is a heterogeneous epithelial tumor invading the oral cavity. It is commonly treated using PD-1 and CTLA-4 inhibitors, but many patients experience resistance to these therapies despite their effectiveness. The OSCC tissue has an expansion of CD4+ cytotoxic lymphocytes (CTLs). These CTLs express the CXCL13 gene, a chemoattractant found to induce a population of double-negative B-cells found to be present in multiple tumor lesions. Additionally, the CXCL13 gene expresses inhibitory receptors such as PDCD1, CTLA4, LAG3, and HAVCR2, which assist in tumor microenvironment suppression, allowing for OSCC persistence [119].

### 4.4. Pemphigus Foliaceus

Pemphigus Foliaceus (PF) is a rare autoimmune disease of the skin, causing blisters and erosions of the epidermis. The disease is mediated by the attack of the Desmoglein 1 protein (DSG1), which is critical for skin adhesion in the upper layers of the epidermis. When this protein is attacked, these layers of skin lose adherence and become separated resulting in blistering of the skin. A similar disease, Pemphigus Vulgaris (PV), attacks the DSG3 protein that facilitates skin adhesion. Both Pemphigus Foliaceus and Pemphigus Vulgaris showed a similar immune repertoire [120]. Heavy chain variable regions of the autoantibodies raised against the DSG proteins in PF serve as a specific target for therapy [82]. Rituximab, a B-cell depletion therapy, is often used for treatment. These Rituximab-treated patients maintain the ability to mount a robust response to vaccination and reconstitute their B-cell population despite their B-cell depletion [120]. Also, there was a marked decrease in DSG autoantibodies with treatment [83].

Immune mechanisms include B-cell clonal expansion with restricted BCR diversity, suggesting an active immune response. Ectopic lymphoid-like structures (ELSs) are found in the skin and mucosal lesions of these patients. These structures mimic secondary lymphoid organs and allow B cell differentiation, clonal expansion, and activation that result in disease persistence [84]. Patients also exhibit a longer CDR3 gene sequence of their IgG autoantibody B-cells with specific IGHV gene segments such as IGHV3-30 [121].

### 4.5. Psoriasis

Psoriasis is a complex autoimmune inflammatory skin disease resulting in epidermal hyperplasia plaques on the body or scalp. Genetic and environmental components are found to be linked to psoriasis outbreaks. The HLA gene is associated with the inheritance of psoriasis. Specifically, HLA-C*06:02 has been identified as the main genetic risk factor for psoriasis. These alleles are involved in CD8+ T-cell antigen presentation which mediates the immune response of psoriasis. HLA-C*06:02 presents the ADAMTS-like protein 5, which is recognized by autoreactive T-cells and results in cytokine production, including IL-17A, IL-22, and IFN-γ that leads to hyperplasia of the skin and chronic inflammation [85]. Regarding T cell repertoire, psoriasis patients displayed a unique TCR oligoclonality of Vβ-Jβ combination of the CDR3 of all defined by the presence in 3 to 5 Vβ subfamilies of a single predominant CDR3 size. However, no significant difference in these Vβ subfamilies was found in the psoriasis lesions compared to the peripheral blood [122]. The absence of the tumor necrosis factor (TNFAIP3) gene was linked to inflammation in psoriasis. Downregulation of this gene results in activation of the p38 MAPK pathway, which in turn increases secretion of IL-17 and TNF-α byTh1 and Th17 and drives the inflammatory process in psoriasis [123].

### 4.6. Vitiligo

Vitiligo is an autoimmune disease characterized by the loss of pigmentation within patches of skin. In biopsy, these patches of skin show complete loss of epidermal pigmentation with the absence of melanocytes at the basal layer and subtle perivascular dermatitis with the presence of CD8+ and CD3+ T cells alongside CD20+ B-cell absence [124]. Nucleotide-binding domain and leucine-rich repeat-containing protein (NLRP) is an innate response immune regulator expressed in Langerhans cells in the skin. The presence of this regulator is associated with increased IL-1β processing, which causes chronic low-level inflammation of the microenvironment, allowing for autoimmune attacks involving the eradication of melanocytes [86]. An NLRP1 variant was found to be contributing to the disease’s chronic inflammation. Multiple TCR clones were found in Vitiligo patients with specific TCRβ repertoires of CD8+ T-cells [86]. The TCR immune repertoire was found to have a specific recombination set of TRBV/TRBJ genes in patients relative to healthy individuals [86]. Current biological therapeutic treatments for Vitiligo include systemic glucocorticoids, phototherapy, and systemic immunosuppressants; however, certain biological therapies have been found to induce Vitiligo. In one study, patients undergoing Alemtuzumab treatment were found to develop vitiligo due to the depletion of B and T-cells from the drug [124]. This further suggests that B-cell depletion along with T-cell activation are involved in the pathogenesis of Vitiligo.

## 5. Conclusions

In summary, TCR and BCR diversity is a key factor in the immune system’s response to self and non-self-antigens and the development of a variety of autoimmune and dermatologic diseases. Studying TCR and BCR repertoires will enable researchers to further understand the onset and progression of diseases and the immune response initiated. It will help create targeted therapeutic interventions. Advancements in technology and high-throughput sequencing have allowed the unveiling of new immune targets, which will ultimately help create new therapeutics novel interventions.

## Figures and Tables

**Table 1:** Immune Repertoire Findings in Autoimmune Diseases.

Disease	Author/Year	Study Design	# of Patients (n)	Immune Repertoire Findings
**Ankylosing** **Spondylitis**	Faham et al. [18]	Prospective	461	14 motifs were found to have association with the HLA-B*27 allele.
	Komech et al. [19]	Cross-Sectional	28	Expansion of TCR-CD23 showed motifs were found to have association with HLA-B27.
	Hanson et al. [20]	Prospective	47	Expansion of TCR-CD3 motif and association of HLA-B27 allele.
	Zheng et al. [21]	Cross-Sectional	21	Expansion of TCR-CD3 motif and association of HLA-B27 allele.
	Komech et al. [22]	Prospective Study	2	Clonal expansion and depletion cause dysbiosis and allow for a relapsing pattern of disease. HSCT may be a possible treatment option.
**Autoimmune Encephalomyelitis**	Litwak et al. [23]	Prospective Study	Murine Study	The mean clinical score and cumulative score of EAE in ngr1−/− mice were reduced but not significantly compared to controls. EAE-induced ngr1−/− and WTLM (control) mice produced similar levels of cytokines splenocyte culture analysis.
	Reddy et al. [24]	Prospective Study	Murine Study	Depletion of CD25+ cells in B10.S (EAE-resistant) mice immunized with PLP 139-151 resulted in approximately one third developing clinical EAE and revealed inflammatory foci in the meninges and brain parenchyma, unlike the control group.
	Schuldt et al. [25]	Prospective Study	Murine Study	Compared to wild-type mice, peripheral γδ T-cell levels were elevated in TCRα+/− β+/− mice.
	Feng et al. [26]	Prospective Study	21	No significant difference in CDR3 amino acid distributions among anti-NMDARE, anti-LGI1E, and HC groups. No significant difference in clonal expansion of each isotype between anti-NMDARE, anti-LGI1E, and HC groups, according to the Gini coefficient comparison per isotype.
**Inflammatory Bowel Disease**	Foth et al. [27]	Systematic Review	N/A *	Lower clonotype diversity and greater clonal expansion were observed in intestinal TCR repertoires compared to the periphery. Though no unique TCR clones were documented, a decrease in CDR3β length and altered hydrophobicity in T-cells was recorded.
	Li, J, et al. [28]	Prospective Study	9	Treg TCR β chain CDR3 repertoire diversity was significantly higher in the spleen compared to the intestine in all patient samples. High-frequency usage of TRBV and TRBJ genes, including TRBV19-01 and TRBJ02-07, was observed in the spleen and intestine across all samples.
	Lopez-Hernandez et al. [29]	Prospective Study	84	Inhibitory KIR2DL5 was recorded more frequently in IBD patients than in healthy controls. No significant difference in HLA-C distribution between healthy controls and patients with IBD.
**Juvenile Idiopathic Arthritis**	Copland et al. [30]	Systematic Review	N/A *	Treg cells in the synovial fluid of JIA patients exhibit more restricted and oligoclonal repertoires compared to those in peripheral blood.
	Morbach et al. [31]	Prospective Study	31	B-cells accumulated in the synovial joints, CD27+IgD− and CD27−IgD− B-cells expressed hypermutated and immuno-switched immunoglobulins.
	Sabbagh et al. [32]	Prospective Study	5	T cell repertoires of JIA patients have significantly increased clonal unevenness, greater variability over time, and fewer IFNγ-producing RS/RA clonotypes compared to healthy controls.
	Spreafico et al. [33]	Prospective study	99	Synovium samples of JIA patients were enriched with circulating pathogen-like lymphocytes, which exhibited lower TCR diversity, similar to synovial T-cells, and produced significantly higher levels of pro-inflammatory cytokines, including interferon-γ, interleukin-17, and tumor necrosis factor-α.
	Wu et al. [34]	Prospective Study	5	Immediately following ASCT, decreases in the frequencies of CD3+ T-cells and CD19+ B-cells, along with an increase in the frequency of CD14+ monocytes, were observed. CDR3 length distribution was predominantly oligoclonal after ASCT but was fully restored 12 months post-ASCT.
**Multiple Sclerosis**	Palanichamy et al. [35]	Prospective Study	8	Peripheral CD27+IgD− memory B-cells, CD27hiCD38hi plasma cells/plasmablasts, and CD27−IgD− B-cells were all detected as having connections to CSF in 6 of 8 patients.
	Greenfield et al. [36]	Prospective Study	10	CD19+CD27+IgD− Ig class-switched memory B-cells were enriched in the CSF of patients compared to the peripheral blood. Persistent CSF Ig-VH clusters across both study time points were represented in 5 out of 10 study patients.
	Stern et al. [37]	Prospective Study	5	Total of 9 clonal lineages present in both the CNS and peripheral secondary lymphoid tissue (draining cervical lymph nodes) were detected.
	Lomakin et al. [38]	Prospective Study	15	Frequency of IGLV1-44 and IGLV3-21 germline genes is elevated in all MS study patients; the difference is statistically significant between highly active MS patients and healthy donors. IGKV2D-29, IGKV3D-20, and IGKV6-21 genes are more commonly found in MS study patients than in healthy donors. IGHV2-26, IGHV2-5, and IGHV2-70 heavy chain germlines were absent in MS patients.
	Gottlieb et al. [39]	Prospective Study	5	TCRβ CDR3 clonality in cells responding to brain stimulation was similar to cells responding to EBV, VZV, or flu. TCR repertoire of cells responding to a specific antigen showed minimal similarity to the repertoire of cells from the same individual responding to different antigens.
**Myasthenia Gravis**	Bernard et al. [40]	Preclinical Animal Trial	35	TCR repertoire of AChR reactive T-cells determines the course of disease.
	Vander Heiden et al. [41]	Cross-Sectional Study	13	MG patients with AChR and MuSK antibodies displayed biased IGHV4 gene segment usage in both V_H_ and V_L_ sequences in the naïve and memory compartments. The memory compartment of patients with AChR antibodies had a decrease in positive selection of somatic mutations in the V_H_ CDR and altered V_H_ CDR3 physicochemical properties. The VL repertoire of MuSK displayed diminished V/J segment distance in recombined sequences, suggesting a reduction in VL receptor editing during B-cell development.
	Lee et al. [42]	Cross-Sectional Study	10	TCR analysis of MG patients shows several specific genes upregulated, a higher abundance of T-cells with longer CDR3 sequences, and overall T-cell clonality was not increased. All these factors contribute to the formation of B cells that create autoreactive antibodies.
	Jiang et al. [43]	Clinical Trial	82	Patients with persistent circulating disease T cells after thymectomy have more severe disease than those without.
**Neuromyelitis Optica**	Afzali et al. [44]	Prospective Study	Murine Study	B-cell-specific ablation of AQP4 restored the number of P41-10- I-Ab+CD4+ single-positive thymocytes to levels seen in global-Aqp4−/− mice; these thymocytes were significantly higher in the thymus of B-cell-deficient mice.
	Kalluri et al. [45]	Prospective Study	Murine Study	Immunization with AQP4 peptides specific to those detected in the natural T cell repertoire of wild-type mice, AQP422–36 and AQP4289–306 specific, did not result in spinal cord disease. However, production of IFN-γ, IL-5, and IL-10 by antigen-specific T-cells occurred following sensitization with AQP4 peptides. Immunization with full-length AQP4 resulted in an AQP4-specific antibody response that included IgG1 and IgG2.
	Miao et al. [46]	Prospective Study	151	Patients with NMOSD had a significantly lower number of unique clones and a reduced proportion of in-frame clones compared to healthy controls. Among identified nonfunctional TCR clones in NMOSD subjects, CDR3 length was significantly shorter and had higher sequence similarity than randomly selected CDR3 sequences.
	Sagan et al. [47]	Prospective Study	Murine Study	Thymic AQP4 deficiency alone was insufficient to completely restore AQP4-reactive T-cells. AQP4-specific Th17 cells induced paralysis in recipient mice, regardless of wild-type or B-cell-deficient status, but all later recovered following apoptosis of donor T-cells. In recipient mice deficient in both T- and B cells or those lacking only T-cells, the donor AQP4-reactive T-cells survived, and paralysis persisted.
**Primary Biliary Cholangitis**	Han et al. [48]	Prospective Study	Murine Study	Predominant CDR3 length of the TCR β-chain is shorter in CD8αα cells compared to CD8αβ cells. The complexity of the TCR β-chain CDR3 region is reduced in CD8αα T-cells.
**Rheumatoid** **Arthritis**	Lu et al. [49]	Prospective Study	7	RF+ and ACPA+ cells were significantly higher in blood from RA patients, compared to healthy controls, though the enrichment was small.
	Heinicke et al. [50]	Prospective Study	37	Trend in higher miR-155-5p expression in blood-derived CD19+ B cells was shown in newly diagnosed RA patients.
	Hu et al. [51]	Prospective Study	164	Proportion of CD27+IgD+ B-cells was significantly lower in the peripheral blood of 31 RA patients. Production of IgM by CD27+IgD+ B-cells was significantly reduced in individuals with RA.
	Wang et al. [52]	Prospective Study	3494	ACPA+ RA patients exhibited significantly higher HLA-DR expression in both naïve and memory B cell populations, whereas ACPA− patients displayed increased HLA-DR expression only in certain clusters. Significant reduction in the frequency of IgA+ B-cells compared to controls in ACPA+ RA patients. RA immunoglobulin sequences consistently exhibited significantly lower SHM than control subjects.
	Scheel et al. [53]	Prospective Study	16	Significant alterations in VH-gene usage were more pronounced in plasma cell aggregates. Increase in CDR3 length was detected in plasma cell VH sequences. RA synovial tissue findings support preferential accumulation of plasma cells and antigen-specific B-cell activation.
	Dunlap et al. [54]	Prospective Study	12	High degree of clonal overlap detected between Tph and T follicular helper (Tfh) clusters. Clonal expansion of Tph and GZMA + CCL5+ memory clusters was identified. Across blood and synovial tissue, the highest clonal expansion among CD4+ T cells exhibited by GNLY+ cytotoxic cluster.
	Ishigaki et al. [55]	Prospective Study	10	Oligoclonal expansion of major memory-ECs (expanded CD4+ T-cell clones) persisted in peripheral blood samples of RA patients.
	Monserrat et al. [56]	Prospective Study	92	Significant increases in CD4+ naïve T (TN), effector memory T (TEM), and CD4+ terminal effector (TE) lymphocytes were detected in recently diagnosed DMARD-naïve RA patients.
	Yang et al. [57]	Prospective Study	994	CDR3 diversity in RA patients was significantly decreased compared to healthy controls. 577 out of 625 V-J gene pairs exhibited a significant frequency difference in the baseline of RA patients compared to that of the control group.
	Taneja et al. [58]	Prospective Study	Murine Study	DRB1*0402 mice exhibited higher numbers of regulatory T-cells compared to DRB1*0401 Tg mice and increased levels of activation-induced cell death. DRB1*0402 mice have lower numbers of thymic T-cells compared with DRB1*0401 mice.
	Repac et al. [59]	Prospective Study	N/A *	42 out of 205 T-cell epitopes related to RA originated from Antigen 1, the highest number of epitopes overall.
	Kramer et al. [60]	Prospective Study	Murine Study	IgM from pSS B-cells salivary exhibited similar charge and hydrophobicity across salivary glands, central lymph nodes, and spleen. IgM from pSS splenic cells had a higher frequency of JH4 usage compared to that from salivary tissue, while salivary IgM exhibited increased JH1 usage relative to central lymph nodes.
**Sjögren’s Syndrome (SjS)**	Meng et al. [61]	Prospective Study	Murine Study	Defect in the early tolerance checkpoint in SjS mice was insufficient to propagate disease in mice with pre-existing autoantibodies (B6.56R). SjS mice crossed anti-dsDNA antibody heavy chain knock-in mice exhibited lower levels of anti-dsDNA antibodies compared to B6.56R mice and reduced salivary gland infiltration relative to SjS model mice.
	Visser et al. [62]	Prospective Study	5	No significant difference in VH-gene usage between intraductal and periductal B-cells. 32 clones were identified among both intraductal and periductal B-cells. Total of 12 IGHV rearrangements encoding rheumatoid factors (RF) were observed and derived from both intraductal B cells and periductal B-cells.
	Voigt et al. [63]	Prospective Study	29	TCR motif analyses indicate that Th1 and Th17 cells possess conserved amino acid sequences in CDR3 regions. Th1 and Th17 cells from patients with pSS display a reduced diversity in V/J pairing. Patients with pSS show a limited usage of TRBJ genes, especially TRBV, with no distinct pairings found at significantly higher frequencies.
	Wanchoo et al. [64]	Prospective Study	Murine Study	Significant increase in IFN-γ and IL-17A-producing effector T-cell in female over male SjS mice Motif analysis of the CDR3 regions showed distinct differences in conserved amino acids between male and female SjS mice.
	Hou et al. [65]	Prospective Study	20	Significant decrease in BCR-H diversity and BCR-H CDR3 length in SLE patients compared to healthy controls. Usage frequency of IGHV3-49, IGHV2-70, and IGHV2-5 genes was significantly different in SLE patients.
**Systemic Lupus Erythematous**	Yuuki et al. [66]	Systematic Review	N/A *	Usage of VDJ genes was significantly skewed in unswitched memory B-cells of SLE. CDR3 length of class-switched memory B-cells and plasmablasts were longer in SLE patients.
	Schleinitz et al. [67]	Prospective Study	13	Compared to healthy controls, SLE patients showed a decreasing trend in NK cell percentage, but it did not reach significance. No difference in expressions of the following receptors in SLE patients compared to controls: NKp46, NKp30, NKp44, CD2, CD69, NKG2A, CD158a/h, CD158e1/e2 and CD158i.
**Systemic Sclerosis**	Shi et al. [68]	Cross-Sectional Study	12	CDR3 length is found to be shorter, and clonotype diversity is greater with disease.
	Servaas et al. [69]	Prospective Study	4	TCRβ type cell persistence is associated with fibrotic deposition in SS.
**Thyroid Disease**	Cui et al. [70]	Cross-Sectional Study	12	Patients with PTC and Hashimoto’s, there is a significant increase in CD3+, CD4+, and CD8+ T-cells.
	Jia et al. [71]	Cross-Sectional Study	12	Expansion of TCR repertoire and several specific genes were found to be associated with Hashimoto’s and Grave’s disease.
	Martin et al. [72]	Translational study	N/A *	Higher-affinity clones responsible for Hashimoto’s have a greater degree of hydrophobicity in CDR3 α region amino acids.
	Marrero et al. [73]	Prospective Study	Murine Study	73% of public repertoire in diabetic mice was represented by the five most frequent TRBV genes expressed by public clonotypes: TRBV13-2, TRBV5, TRBV1, TRBV2, and TRBV15. TRBV13-2, TRBV13-1, and TRBV5 genes were expressed by 18.5%, 13.1%, and 9.8% of all unique clonotypes from diabetic mice.
**Diabetes Mellitus**	Jacobsen et al. [74]	Systematic Review	N/A *	Reported greatest risk of developing Type 1 Diabetes is the HLA-DR3/DR4-DQ8 haplotype; during thymic selection, HLA molecules greatly influence TCR repertoire.
	Zhou et al. [75]	Prospective Study	N/A *	According to the retention of CLIP, DQ2, DQ8, or DQ2/8 trans-dimers were more resistant to DM editing than T1D-protective DQ molecules. DQ8, DQ2-8, and DQ8-2 retained high levels of cell surface CLIP in the presence of DM.
	Hanna et al. [76]	Systematic Review	N/A *	Insulin-binding BCRs exhibited skewed usage of JH6 gene segments, which were biased toward positively charged amino acid sequences in CDR3 regions.

N/A *: Patient sample size was not provided in the associated publication.

**Table 2:** Immune Repertoire Findings in Dermatologic Diseases.

Disease	Author/Year	Study Design	# of Patients (n)	Immune Repertoire Findings
**Alopecia Areata**	de Jong, et al. [77]	Cross- Sectional	Murine study	CD8+ NKG28+ cell is suggested to be the dominant effector cell population responsible for hair loss in mice.
	Matthew, et al. [78]	Clinical Trial	31	Therapies targeting reduced clonal expansion of CD8+ T-cells are possible areas of treatment.
**Melanoma**	Kotlan et al. [79]	Basic Translational	N/A *	Melanoma cell lesions contained anti-GD3 ganglioside-binder antibody-variable region genes. DNA sequence analysis revealed an overrepresented VH3-1 cluster, represented in melanoma TIL-B immunoglobulin repertoire. Antibody fragments showed binding potential to disialylated glycosphingolipids and their O-acetylated forms on melanoma cancer cells.
	Godet et al. [80]	Prospective Study	9	All clones derived from melanoma patients were reactive against the MELOE-1(36-44) peptide and against HLA-A2(+) melanoma cell lines, suggesting a large TCR repertoire specific to the MELOE-1/A2 epitope.
	Simon et al. [81]	Prospective Study	10	A highly conserved “GP” motif in the TRAV19 chain, possibly involving the TRBJ2-1 segment. In the CDR3α region for MELOE-1, specific T cell repertoire was identified. The analysis of T-cell clonotypes from melanoma patients revealed the existence of public CDR3α and ß clonotypes for Melan-A and MELOE-1 specific T-cells.
**Pemphigus Foliaceus**	Mouquet et al. [82]	Prospective study	21	Rituximab depleted autoreactive B-cells, leading to the elimination of anti-Dsg autoantibodies in most remitted patients and the re-emergence of a diverse B cell repertoire by naïve B lymphocytes. One third of these patients expressed a transitional CD19+CD38 (high)CD24(high) phenotype.
	Zhou et al. [83]	Retrospective study	97	B-cell receptor repertoire analysis revealed the clonal expansion of the lesional B-cells and significantly increased Dsg1-or Dsg3-specific CD19+ B-cells in lesions than in peripheral blood. In contrast, the frequency of CD19+CD27 IgD+ naïve B-cells was much lower in the pemphigus lesions than in the peripheral blood.
	Calonga-Solis et al. [84]	Prospective Study	30	Lower B cell clonotype diversity in the B cell immune repertoire of patients and controls from the endemic area, suggesting the immune repertoire. Longer CDR3 sequences and differential disease-specific usage of IGHV segments (increased IGHV3-30 and decreased IGHV3-23) were found in patients. Findings suggest environmental factors in PF can impact the diversity of the repertoire.
**Psoriasis**	Bour et al. [85]	Prospective Study	10	Psoriasis patients displayed a unique TCR oligoclonality of Vβ-Jβ combination of the CDR3 of all defined by the presence in 3 to 5 Vβ subfamilies of a single predominant CDR3 size.
**Vitiligo**	Xiong et al. [86]	Cross-Sectional Study	9	TCR immune repertoire was found to be less heterogeneous as a specific recombination set of TRBV/TRBJ genes could differentiate vitiligo patients.

N/A *: Patient sample size was not provided in the associated publication.

## Data Availability

No new data were created in the study.
